# Detection of fast oscillating magnetic fields using dynamic multiple TR imaging and Fourier analysis

**DOI:** 10.1371/journal.pone.0189916

**Published:** 2018-01-10

**Authors:** Ki Hwan Kim, Hyo-Im Heo, Sung-Hong Park

**Affiliations:** 1 Department of Bio and Brain Engineering, Korea Advanced Institute of Science and Technology, Daejeon, South Korea; 2 Graduate School of Medical Science and Engineering, Korea Advanced Institute of Science and Technology, Daejeon, South Korea; University of Queensland, AUSTRALIA

## Abstract

Neuronal oscillations produce oscillating magnetic fields. There have been trials to detect neuronal oscillations using MRI, but the detectability in *in vivo* is still in debate. Major obstacles to detecting neuronal oscillations are (i) weak amplitudes, (ii) fast oscillations, which are faster than MRI temporal resolution, and (iii) random frequencies and on/off intervals. In this study, we proposed a new approach for direct detection of weak and fast oscillating magnetic fields. The approach consists of (i) dynamic acquisitions using multiple times to repeats (TRs) and (ii) an expanded frequency spectral analysis. Gradient echo echo-planar imaging was used to test the feasibility of the proposed approach with a phantom generating oscillating magnetic fields with various frequencies and amplitudes and random on/off intervals. The results showed that the proposed approach could precisely detect the weak and fast oscillating magnetic fields with random frequencies and on/off intervals. Complex and phase spectra showed reliable signals, while no meaningful signals were observed in magnitude spectra. A two-TR approach provided an absolute frequency spectrum above Nyquist sampling frequency pixel by pixel with no *a priori* target frequency information. The proposed dynamic multiple-TR imaging and Fourier analysis are promising for direct detection of neuronal oscillations and potentially applicable to any pulse sequences.

## Introduction

Functional MRI (fMRI) based on blood-oxygenation-level-dependent (BOLD) contrast was introduced more than 20 years ago, and now has become a dominant tool for mapping brain function noninvasively [1–3]. Further, resting-state fMRI has been widely used to uncover the “functional connectivity” of the resting-state brain [4, 5]. However, BOLD signals originate from veins, which are affected by secondary hemodynamic responses associated with local neuronal activity [6–9]. To overcome the limitation of BOLD fMRI, many researchers have tried to use MRI for more than 15 years to directly detect magnitude or phase signals produced by transient magnetic fields. Phantom experiments demonstrated that MRI can detect weak magnetic fields in the order of 0.1−1 nT [10–13]. However, many research groups have reported both positive [12–16] and negative results [17–22] in *in vivo* studies, and a consensus has not been reached on the detectability of the neuronal currents *in vivo*.

Recently, a method based on the spin-locking mechanism, named stimulus-induced rotary saturation (SIRS), was introduced to resonate the B_1_–induced rotation with neuronal oscillations [23]. The sensitivity has been improved with a modified SIRS technique, which detected oscillating magnetic fields with < 1 nT in a phantom study [24]. The same research group tried *in vivo* studies using the modified SIRS technique, but failed to detect the neuronal currents *in vivo* [24]. Such failures of *in vivo* imaging suggest that there are problems other than the sensitivity of the MR methods.

Understanding the characteristics of neuronal oscillations is crucial for imaging neuronal activity. Based on this, several factors should be considered for *in vivo* experiments. First, neuronal oscillations are faster than the temporal resolution of MRI. Since high frequency oscillations such as gamma oscillation cannot be sufficiently captured even by a fast technique (e.g. EPI), previous experiments using EPI mostly focused on slow alpha waves [19, 25]. Recently, signals from non-BOLD sources with frequencies above 0.5 Hz have been investigated with resting-state fMRI, however, the frequency of interest is still limited [26–32]. When neuronal oscillations are sampled at a rate insufficient to capture the up/down of the oscillations, aliasing occurs according to the Nyquist theorem [33]. Therefore, a new analytical strategy is necessary for evaluating insufficiently sampled data to detect fast neuronal oscillations. Second, a previous study based on magnetoencephalography (MEG) experiments indicated that the magnetic fields generated by spontaneous neuronal oscillations (order of 1 nT) can be much stronger than those generated by evoked potentials (order of 0.1 nT) [34]. Nonetheless, most previous studies using MRI have focused on evoked potentials by stimulation, because it is easily synchronized to the MR sequence [11, 12, 35, 36]. However, spontaneous neuronal oscillations cannot be synchronized with the MR sequence, and thus it is necessary for the new data acquisition and analysis to be independent of the synchronization with the neuronal oscillations. Lastly, there are synchronous and asynchronous neuronal oscillations [37, 38]. The detectability of synchronous oscillations can potentially be enhanced by temporal averaging, which may be the only way to detect the weak magnetic fields generated by neuronal oscillations. Sufficient average will also help imaging/analysis methods to be less sensitive to slight variations in phase and frequency of neuronal oscillations.

In this work, we proposed a new data acquisition / analysis strategy, which consists of dynamic multiple-TR imaging and Fourier analysis, to detect weak magnetic fields oscillating at a frequency above the Nyquist sampling frequency of MRI temporal resolution. We tested its feasibility using gradient echo echo planar imaging (GE-EPI). We described the proposed approach in detail and validated it using simulations and phantom experiments. We also discussed potential advantages and limitations of the proposed approach for *in vivo* imaging.

## Methods

### Theory

**Fig 1:**
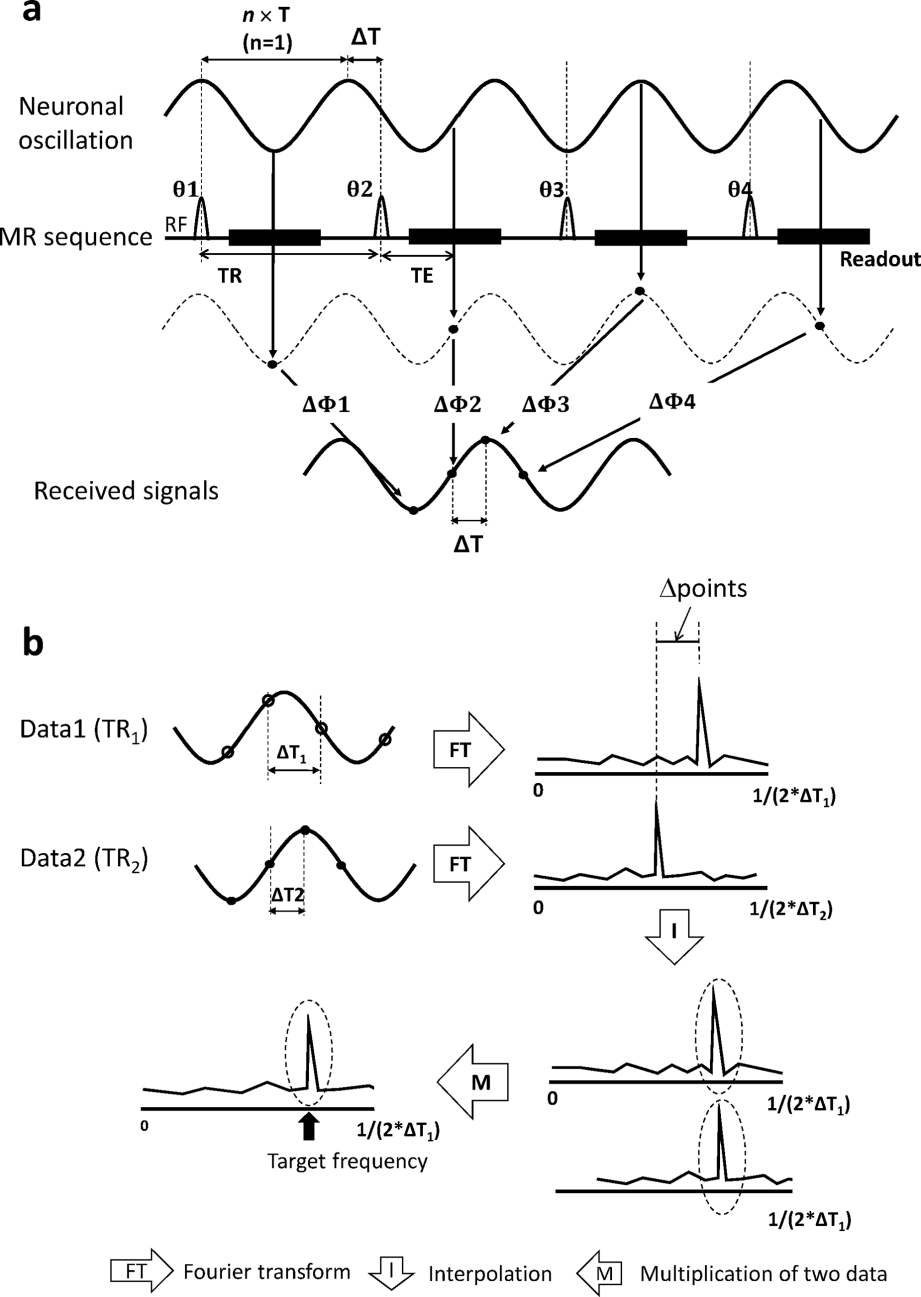
Diagrams explaining the proposed approach. **a:** Dynamic MR acquisition with one TR value. Even when the sampling rate is lower than twice the frequency of oscillating magnetic field, oscillating signals can be captured at different phases (θ = θ_1_, θ_2_, θ_3_ ···) between the oscillating magnetic field and the MR sequence. As the MR phase signals (ΔΦ = ΔΦ_1_, ΔΦ _2_, ΔΦ _3_ ···) depend on the relative phase (θ), the oscillating magnetic field can be encoded as oscillating phase signals (ΔΦ) in the dynamic MR acquisition. **b:** Two−TR approach. The oscillating magnetic field was captured with two different sampling periods (ΔT_1_ and ΔT_2_) in two TR datasets (TR_1_ and TR_2_). Two frequency spectra were derived by applying 1D Fourier transform to two TR datasets. In order to match number of points and the frequency range of the two frequency spectra, linear interpolation was applied to the frequency spectrum from the smaller sampling period (data 2 in this figure). Next, the target frequency components (dotted circle) in two frequency spectra were multiplied and then the response at the target frequency was selected. The residual time ΔT, which is determined from TR and T, acts as a sampling period in the oscillating phase signals (ΔΦ). T: period of the neuronal oscillation, TR: repetition time, TE: echo time, and *n*: the closest integer multiple of T to TR.

The proposed approach consists of two steps: (i) dynamic data acquisition using multiple times to repeats (TRs) and (ii) an expanded frequency spectral analysis. Fig 1 shows the diagram of insufficient sampling of the oscillating magnetic field with the frequency of interest, hereafter referred to as the target frequency. When the target frequency is higher than a half of the sampling rate, the target frequency component is insufficiently captured, resulting in aliasing. Such aliased signals are encoded by manipulating the phase offset (*θ*) between the oscillating magnetic field and the MR sequence, where the residual time *ΔT* acts as the sampling period in the aliased signal (Fig 1A). The target frequency component can be evaluated by 1D Fourier transform using the sampling period *ΔT*. However, the aliased signals cannot be distinguished from fully sampled low frequency signals. To overcome the problem, a data acquisition strategy using two different TRs is proposed. Using two frequency spectra acquired with the two TRs, the low and high frequency components can be precisely reconstructed in a new spectrum (called an “absolute frequency spectrum” throughout this paper).

More specifically, as the transverse magnetization generated by excitation RF pulses is affected by the oscillating magnetic fields during TE, a phase signal (*ΔΦ*) is added to the MR signal (Fig 1A).

$$\Delta \Phi =\int_{0}^{TE}\gamma \Delta B_{z}(t)\sin(2\pi f_{0}\cdot t+\theta )dt$$ (1)

where *f*_0_ represents frequency of the oscillating magnetic field and *θ* represents the relative phase offset between the oscillating magnetic field and the MRI sequence. The MR phase signal (*ΔΦ*) is oscillating across dynamic MR images, when the phase offset *θ* is manipulated to increase in a constant value. Here effective sampling period of the phase signal (*ΔΦ*) is equal to the residual time (*ΔT*), which is derived from TR divided by the period of the oscillating magnetic fields (*T*), as follows.

TR=n×T+ΔT(ΔT<T) (2)

where *n* is the closest integer multiple of *T* to TR.

Since the effective sampling period *ΔT* depends on the target frequency, the peak only at the target frequency can be precisely evaluated. Also, the peak at the target frequency cannot be differentiated from the sufficiently captured low-frequency signals in a single-TR experiment. A two-TR approach is proposed to overcome the problem (Fig 1B). Difference in TR leads to difference in the sampling period *ΔT*, as described in Eq (2). In the two-TR approach, the target frequency component (*f*_0_) will be relocated as described by

$$\frac{\Delta po\mathrm{int}}{N}=\frac{f_{0}}{1/\Delta T_{1}}-\frac{f_{0}}{1/\Delta T_{2}}=f_{0}(\Delta T_{1}-\Delta T_{2})=f_{0}\Delta TR$$ (3)

where *Δpoint* is the number of points in which the target frequency component was relocated between the two spectra, N is the number of total samples, *ΔT*_*1*_ and *ΔT*_*2*_ are the effective sampling periods of the two TRs, and ΔTR is the difference in TRs (Fig 1B). The Eq 3 is valid under the assumption that *n* values of the two TRs in Eq 2 are the same. Since *Δpoint* depends on the target frequency (*f*_*0*_), the aliased signals can be distinguished from the sufficiently captured low-frequency signals using the two spectra. By taking advantage of the information from the two-TR spectra, we can evaluate the multiple frequency components precisely in an absolute frequency spectrum, as follows (Fig 1B). A specific target frequency was selected from the range of [$-\frac{1}{2\Delta TR},\frac{1}{2\Delta TR}$]. The target frequency and the two TRs determined the sampling periods (Δ*T*_1_, Δ*T*_2_) based on Eq 2. The spectrum from the shorter sampling period of the two (i.e., the larger sampling frequency) was scaled down by linear interpolation to match the spectrum from the longer sampling period in terms of number of points and frequency range. The two spectra were then multiplied to each other and the value at the target frequency was selected from the multiplied spectrum as the response at the target frequency. This procedure was repeated for all the target frequency candidates in the range of [$-\frac{1}{2\Delta TR},\frac{1}{2\Delta TR}$], to compose the new absolute frequency spectrum, where all frequency components could be assessed precisely without aliasing.

### Simulations

A K-space was generated by 2D Fourier transform of the Shepp-Logan phantom image from the MATLAB built-in function. The generation of K-space was repeated to compose dynamic K-space data, where the oscillating magnetic field *ΔB*(*t*) of 1-nT amplitude and 25-Hz frequency parallel to the main magnetic field was applied. Then, 2D Fourier transform converted the dynamic K-space data into complex, phase, and magnitude image sets. Random noise with normal distribution and standard deviation of 0.05 was added to the images. In order to evaluate the temporal averaging effects, the number of dynamic images was varied to be 200, 500, and 1000. Another simulations were conducted with *ΔB*(*t*) turned on/off in random time intervals, where the relative phase offset (*θ*) was repetitively changed.

### Phantom preparation

A single loop coil made of 26-gauge copper wire coated with an insulation layer was wound around a plastic tube with 1.6-cm diameter. The tube was filled with gadolinium-based contrast agent solution whose T_1_ and T_2_ were 701ms and 460ms, respectively. The magnetic field *ΔB* generated by the coil was parallel to *B*_*0*_. The field strength was calculated at the center of the coil using Biot-Savart law. Currents with sinusoidal waveform were produced using a function generator (33210A, Agilent, Santa Clara, CA) and applied to the phantom through a resistor (2.4kΩ).

### Phantom experiments

All experiments were performed on a 3-Tesla MRS 3000 scanner with 17-cm bore size (MR Solutions, Surrey, United Kingdom) with a birdcage mouse body coil. GE-EPI experiments were performed with and without the stimulations separately, to distinguish stimulation-induced peaks from systematic noises. Since the proposed method was designed to detect neuronal oscillations faster than alpha waves (~10Hz), the sensitivity to magnetic fields oscillating at a randomly-chosen frequency of 25 Hz was evaluated at varying magnetic field strengths (*ΔB*) of 0.5, 1, 5, and 10 nT with dynamic acquisition of 1000 images per each *ΔB*. Using stimulation with *ΔB* = 1nT, temporal averaging was tested using different numbers of dynamic images from 1000 to 5000 with 1000 step. Multiple-TE experiments were performed with TE varying from 20 to 55 ms with 5-ms step at various stimulation frequencies of 25, 30, and 35 Hz. At each condition of TEs and stimulation frequencies, 2000 dynamic images were acquired. Default amplitude and stimulation frequency were 5nT and 25Hz, respectively, unless specified otherwise. The default parameters for GE-EPI were: TR = 90 ms, matrix = 64×64, field of view = 5×5 cm^2^, slice thickness = 2 mm, number of slice = 1, number of scans = 1000, TE = 20ms, and flip angle = 11°.

To evaluate the effects of the random phase changes on the detection of the magnetic field oscillations, the stimulation was randomly turned on and off during the acquisition of 1000 dynamic images. Total time intervals of the ON state were approximately the same as those of the OFF state. To simulate random changes in frequency, the stimulation frequency was changed from 25 to 15 Hz after acquisition of the initial 200 images, and then changed back to 25Hz after acquisition of the additional 500 images.

To acquire the absolute frequency spectrum where multiple frequency components could be evaluated precisely with no *a priori* information of oscillation frequencies, the two-TR experiments were performed for stimulations with (i) one frequency component of 25Hz and also with (ii) two simultaneously-applied frequency components of 10 and 15Hz, which were randomly selected. Total 1000 and 2000 dynamic images were acquired in (i) and (ii), respectively. Two TRs of 90 and 91ms were alternatingly applied during every 200 TRs. All dynamic images were classified according to their TR values. The absolute frequency spectrum was obtained by scaling of the two spectra along the frequency dimension based on a frequency of interest, multiplying the two spectra after a linear interpolation, selecting the value at the frequency of interest, and then repeating these procedures in the frequencies of interest ranging from 1 to 50 Hz with 1-Hz step.

### Data analysis

All data analyses were performed using MATLAB (Mathworks, Natick, MA). Complex, magnitude, and phase datasets were derived by applying 2D Fourier transform to the dynamic K-space data. Here “magnitude” was absolute values of the complex images, “phase” was phase angles of the complex images, and “complex” was complex images themselves. Pixel-based 1D Fourier transform converted three dynamic datasets into frequency spectra separately. The frequency range of the spectra was determined by the residual time *ΔT*, which was derived from the target frequency and TR (Eq 2). Background noise bands were selected from each frequency spectrum, and then the spectrum was (i) subtracted by the mean value of the background noise bands, and then (ii) divided by standard deviation of the background noise bands. The former allowed better suppression of systematic noises in the two-TR analysis and the latter converted the spectrum into an SNR spectrum enabling quantitative comparison between complex, magnitude, and phase spectra. The two spectra from the two-TR experiment were combined to generate the absolute frequency spectrum, as described above. A map of signal to noise ratio (SNR) was composed by taking the SNR value at the stimulation frequency in each pixel. A region of interest (ROI) was manually drawn to cover more than 80% of the phantom area, and mean SNR was calculated by averaging the SNR spectra in ROI.

## Results

### Simulations

**Fig 2:**
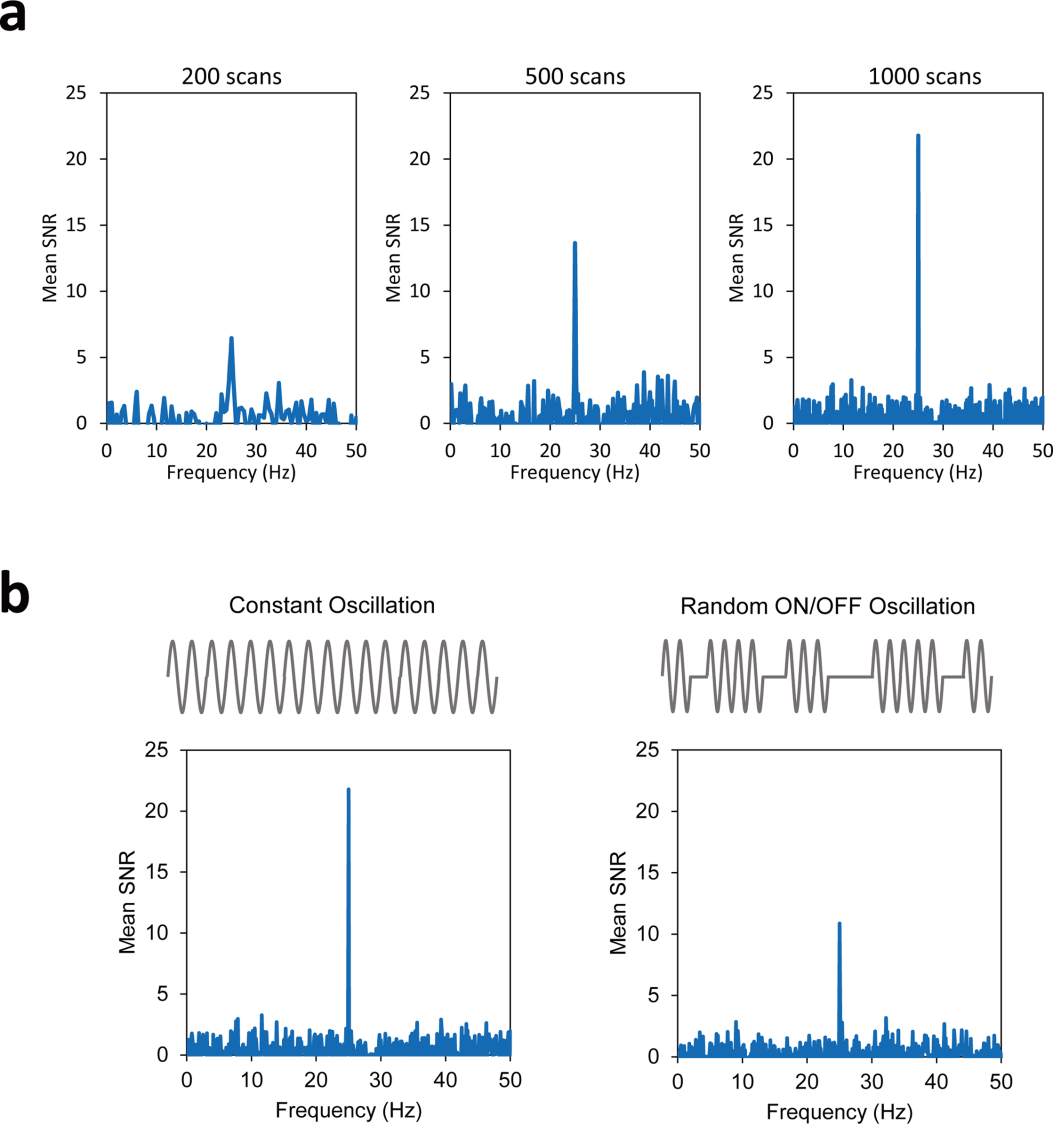
Simulation results for sensitivity of the proposed approach to high-frequency oscillating magnetic field. **a:** Effects of the number of dynamic scans (200, 500, and 1000). Oscillating magnetic fields ΔB(t) with strength and frequency of 1nT and 25 Hz were used. Frequency range of the spectra was adjusted to the sampling period of 10 ms, which was derived from TR of 10 ms and the target frequency of 25 Hz. Mean SNR spectra were derived by ROI averaging. **b:** Comparison of constant (left) and random ON/OFF (right) oscillating magnetic fields with a frequency of 25 Hz. In random ON/OFF oscillation, ΔB(t) was alternatingly turned on and off and the off-state was about 36% of the total states.

The simulation studies showed that the phase signals produced by 25-Hz stimulation (above Nyquist sampling rate) could be detected at 25 Hz in the frequency spectrum adjusted based on the sampling period (*ΔT*) of 10 ms, which was derived from TR of 90 ms and the oscillation period of 40 ms (Eq 2) (Fig 2). The peak detected at 25 Hz showed higher SNR with higher number of dynamic scans (Fig 2A), indicating the temporal averaging effects. Even when the oscillating magnetic fields were randomly turned on and off, the peak was still clearly detected, while SNR of the peak was reduced because of the inclusion of the OFF intervals (Fig 2B).

### Phantom experiments

**Fig 3:**
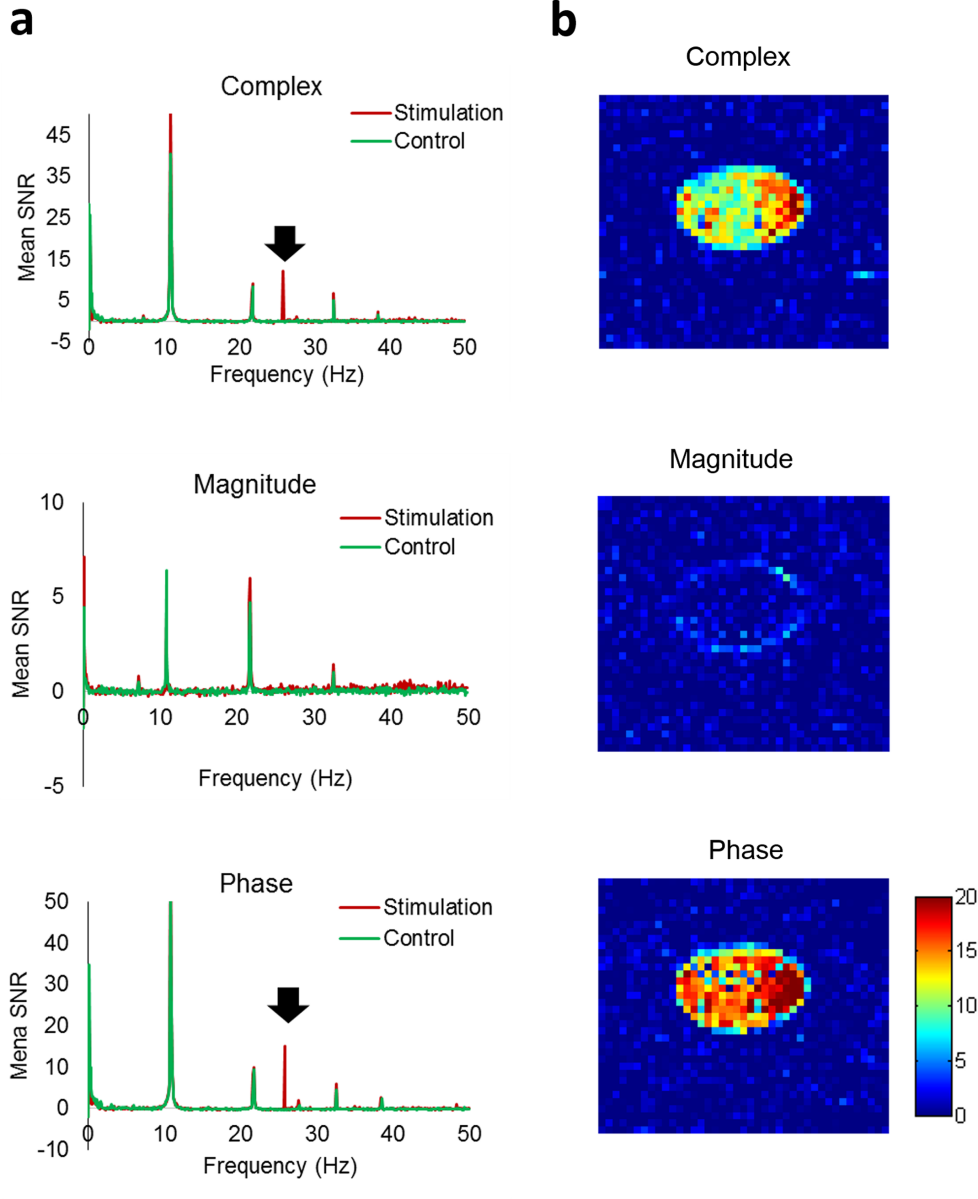
Representative frequency spectra acquired with GE-EPI. The stimulation frequency was 25 Hz. Displayed are frequency spectra with the range adjusted to the residual time ΔT of 10 ms (**a**) and SNR maps at the target frequency of 25 Hz (**b**) for complex (top), magnitude (middle), and phase (bottom) datasets. Each frequency spectrum was acquired in the existence (stimulation) and absence (control) of stimulation (5 nT). The vertical scale of the spectra represents mean SNR of pixels in ROI. Black arrows indicate the peak produced by the stimulation.

**Fig 4:**
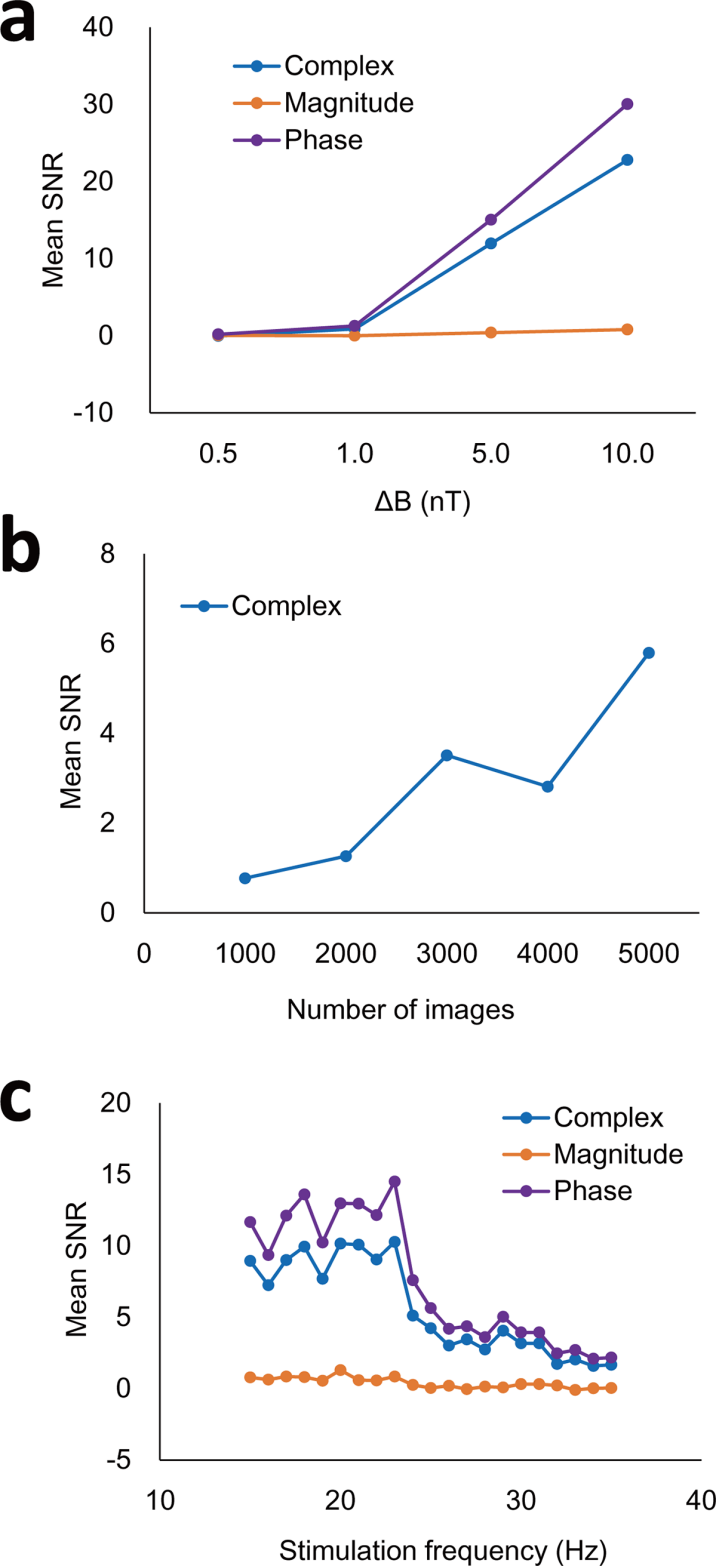
Changes in sensitivity with stimulation strength and scan parameters. Mean SNR of the peak produced by 25-Hz stimulation was evaluated at varying strengths (ΔB(t) = 0.5, 1, 5, 10 nT) (**a**) and at varying numbers of dynamic images from 1000 to 5000 with ΔB(t) = 1 nT (**b**). **c**: Multi-TE experiment. Various stimulation frequencies (= 25, 30, and 35 Hz) were tested at TE values ranging from 20 to 55 ms with a step of 5 ms.

Fig 3 shows representative mean SNR spectra with and without stimulation (25Hz, 5 nT), acquired with the single-TR scan. Based on the sampling period *ΔT* = 10 ms (TR = 90 ms and oscillation period = 40 ms (Eq 2)), a peak was detected at 25 Hz in the stimulation spectra but not in the control spectra (Fig 3A). The peak was observable in the complex and phase datasets, but not in the magnitude dataset (Fig 3A). Several other peaks were also detected in both control and stimulation scans due to systematic noises. SNR maps of 25 Hz showed high signals in most pixels of the phantom (Fig 3B) with heterogeneous distribution presumably due to low spatial resolution or the spiral shape of the coil. SNR of the peak produced by stimulation increased with the stimulation amplitude (Fig 4A). By increasing the number of dynamic scans, SNR could be improved significantly due to temporal averaging effects (Fig 4B), in agreement with the simulation results (Fig 2A). SNR of the peak was also dependent on TE (Fig 4C). Generally SNR decreased with TE due to T_2_* decay. When TE was close to the stimulation period (40, 33, and 29 ms for 25, 30, and 35 Hz, respectively), SNR of the peak was close to zero presumably due to the fact that integration of an oscillating field in one period becomes zero (or a constant value).

**Fig 5:**
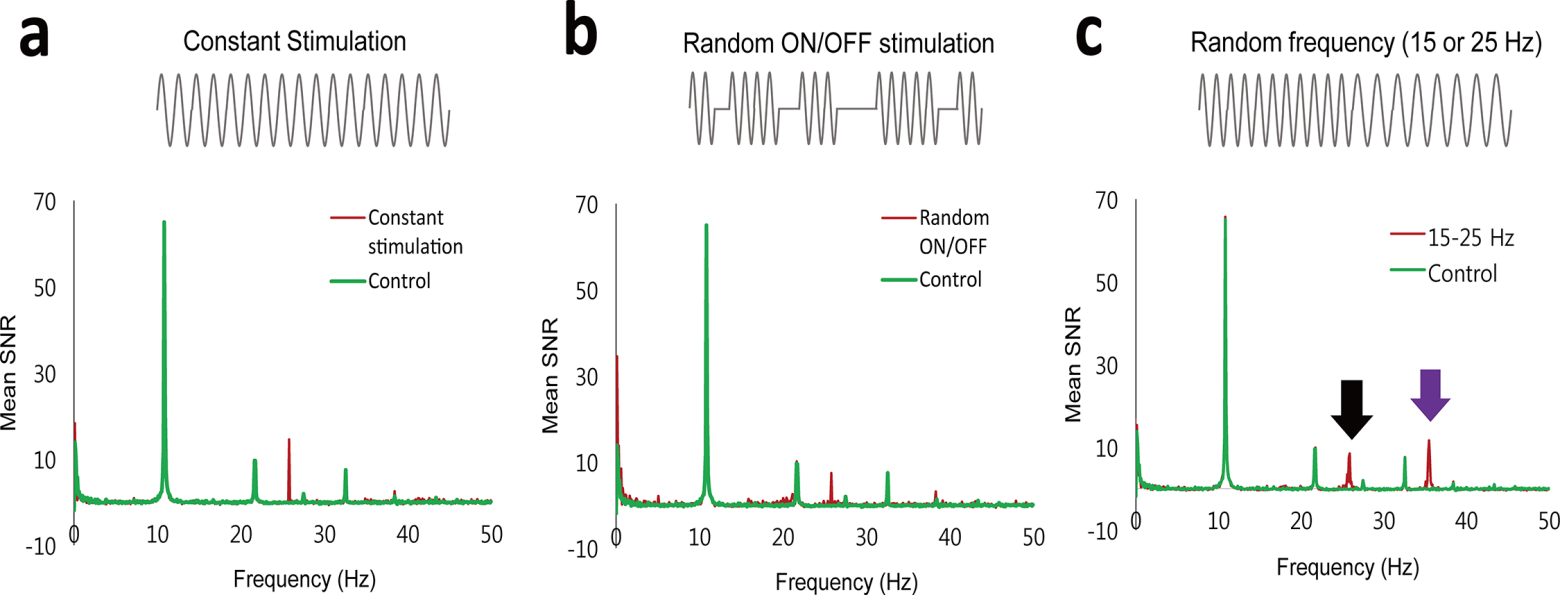
Effects of changes in stimulation frequency and on/off intervals. Frequency range of the spectra were adjusted to the residual time ΔT of 10 ms, which was derived from TR of 90 ms and the target frequency of 25 Hz. **a:** Spectrum acquired with a constant 25-Hz stimulation. **b:** Spectrum acquired with random ON/OFF stimulation. The 25-Hz stimulation was randomly turned on and off. Total lengths of the ON- and OFF-states were about the same. **c**: Spectrum acquired with changes in stimulation frequency. The stimulation frequency was alternated between 15 Hz and 25 Hz repetitively. Black and purple arrows indicate the peaks produced by 25 Hz and 15 Hz. In this single-TR experiment, the peak at 15 Hz was misplaced at 35 Hz on the spectrum when target frequency was set to 25 Hz, which could be resolved in the two-TR experiment.

When the stimulation was manually turned on and off, the peak was still clearly detected, while the SNR decreased due to the inclusion of the OFF state intervals (Fig 5A and 5B), in agreement with the simulation results (Fig 2B). When stimulations with two frequencies of 25 Hz and 15 Hz were randomly alternated, two peaks were simultaneously detected (Fig 5D) but only the peak produced by the 25-Hz stimulation was precisely detected at 25 Hz. Note that in this one-TR experiment the sampling period *ΔT* was adjusted to the target frequency of 25 Hz and thus the peak produced by 15-Hz stimulation was misplaced to appear at 35 Hz (Fig 5B), which could be resolved in the two-TR experiment as described later. It should be also noted that the peak at 25 Hz was lower than the other.

**Fig 6:**
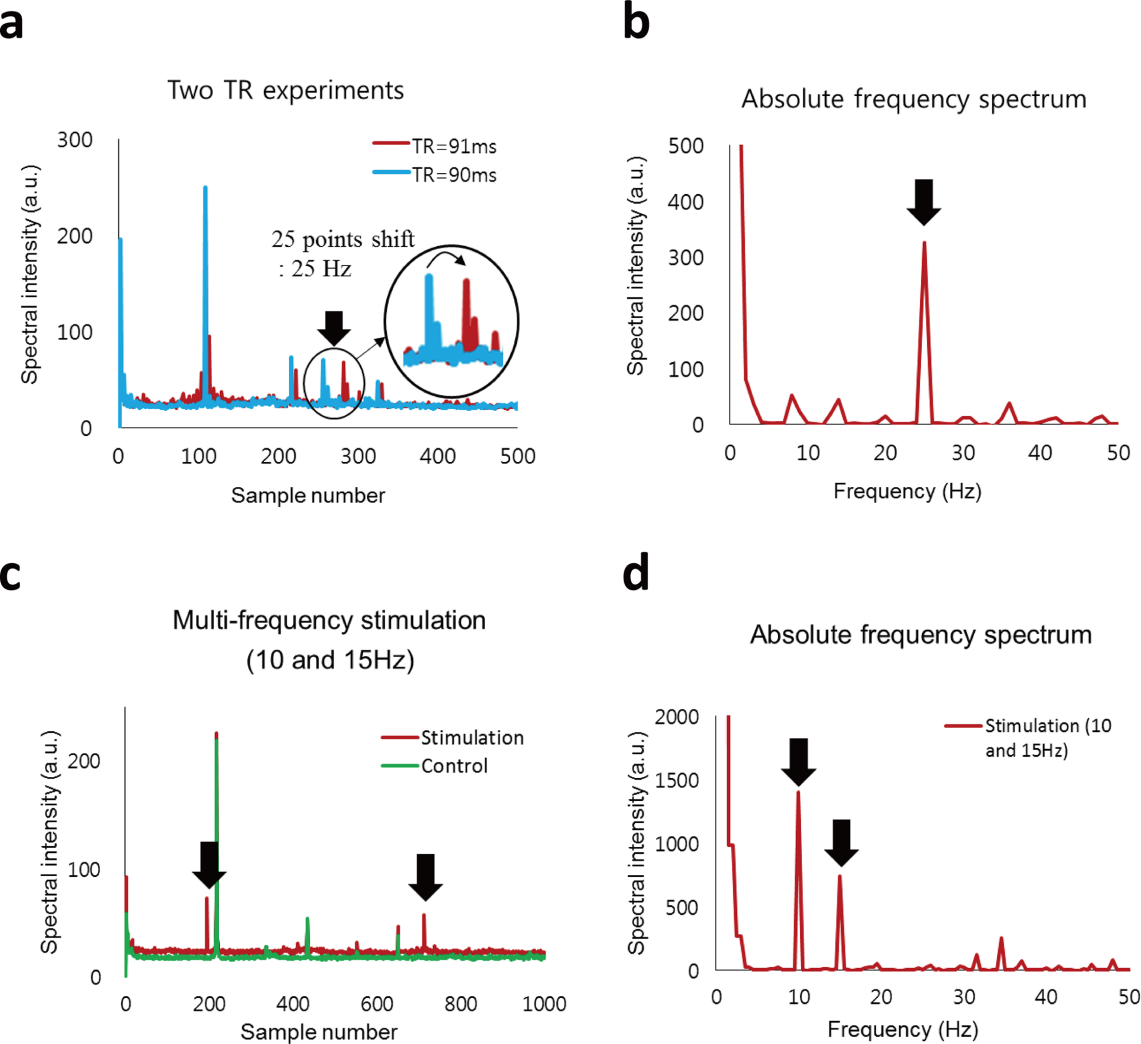
Detection of stimulations with two−TR experiments. **a−b:** Detection of stimulation with one frequency component (25 Hz) using two alternating TRs (TR = 90 and 91 ms). The peak produced by the stimulation (arrow) was shifted in an amount of the stimulation frequency (25 Hz) in the spectrum (**a**) displayed with the sample number in the horizontal direction (**a**). In the absolute frequency spectrum derived from the two-TR experiment (**b**), the peak (arrow) was detected at 25 Hz and systematic noises were suppressed. **c−d:** Detection of multi-frequency stimulation (10 and 15 Hz) using two alternating TRs (TR = 90 and 91 ms). Two peaks (arrows) generated by stimulations were detected in spectrum from one of the two TRs (TR = 90 ms) (**c**). In the absolute frequency spectrum derived from the two-TR experiment, both of the two peaks generated by the stimulation were detected at the right positions (10 and 15 Hz) with no *a priori* target frequency information, while systematic noises were suppressed.

In the two-TR experiments, frequency spectra were separately acquired at two randomly selected TR values (90 and 91 ms) (Fig 6). When TR changed from 90 to 91 ms, the peak produced by the 25-Hz stimulation was shifted by 25 points between the two spectra, as described in Eq 3 (Fig 6A). The absolute frequency spectrum from the two-TR experiments highlighted the peak generated by the stimulation with no *a priori* target frequency information, while suppressing many other frequency components (Fig 6B). The two-TR experiments (90 and 91 ms) were also applied to stimulations with two different frequency components (Fig 6C and 6D). Two peaks were detected at two different locations in each TR experiment (Fig 6C, TR = 90 ms). Point shifts by the TR change (*Δpoint*) were also found to coincide with the amounts predicted by Eq 3, similar to Fig 6A. The absolute frequency spectrum highlighted the two peaks at the right positions (10 and 15 Hz) with no aliasing, while suppressing other systematic noises (Fig 6D). The peak at 15 Hz was lower than that at 10 Hz.

## Discussion

We demonstrated that multiple fast oscillating magnetic fields above Nyquist sampling frequency can be detected in an absolute frequency spectrum with no *a priori* target frequency information through the proposed two-TR acquisition and the expanded Fourier analysis. Both simulations and experiments demonstrated that the proposed method was not affected by various conditions of random changes in frequency and ON/OFF states of oscillating magnetic fields. Furthermore, the proposed method can be potentially implemented in any MRI sequences that have been used for detection of the oscillating magnetic field (e.g., spin-locking methods [23, 24]).

As predictable from Eq 1 under the assumption that Δ*B*_Z_(*t*) is constant, the maximum-possible time-integral (the phase signal, *ΔΦ*) decreases with the target frequency (*f*_0_) (*ΔΦ* ∞1*/f*_0_). This prediction was consistent with our experimental results of the two alternating frequencies (Fig 5C) and the two simultaneously-applied frequencies (Fig 6D). In general, the phase signals from higher target frequencies can be enhanced by adjusting (shortering) TE (Fig 4C), which minimizes the canceling effects between the positive and negative parts of the oscillating magnetic fields.

The goal of the proposed method is to detect synchronous neuronal activities of nearly all or at least a representative fraction of the neuronal population in a voxel. The synchronous neuronal activities contribute to high frequency components of the local field potential [39], and they are considered as potential sources of electrocorticography (ECoG), electroencephalography (EEG), magnetoencephalography (MEG). In ECoG study, a spatial resolution of the recorded electric field is approximately <5 mm^2^ [40]. Considering the spatial resolution of EPI sequence, the potential detectability of neuronal signals within a voxel can be similar to those detected in ECoG. Therefore, the sensitivity of the proposed method is in the level of detecting signals induced by spatially and temporally synchronous neuronal activities, although there may be more complicated confounding factors in real *in vivo* cases.

The approach using frequency spectra was also introduced to detect neuronal oscillations in the previous fMRI studies [13, 16, 19, 25], but the spectrum of interest was limited by TR, i.e., [$-\frac{1}{2TR},\frac{1}{2TR}$]. Since spontaneous neuronal oscillations have a wide frequency range (~200 Hz) [41], the whole range of brain waves cannot be covered even by fast MRI techniques (e.g. EPI). In the proposed approach, the frequency over the Nyquist sampling frequency could be analyzed. The two-TR approach provided the signals from oscillating magnetic fields with no aliasing in the range of [$-\frac{1}{2\Delta TR},\frac{1}{2\Delta TR}$], while suppressing systematic noises. Spontaneous BOLD activity (<0.1 Hz), cardiac pulsation (60-100/min) and respiration (12-18/min) show lower frequencies than those of brain waves [42, 43]. Based on the difference in frequencies between brain waves and other components, the non-neuronal signals may be differentiated by the proposed method.

Several *in vitro* studies showed that MRI can detect the oscillating magnetic field induced by neuronal currents [44, 45]. The phase shifts of 0.15–3°, equivalent to the magnetic field changes of 0.2–3.9 nT, were detected using brain cultures *in vitro* [44]. However, there are also several negative reports. In the study by Luo et al [46], the authors reported detection of no significant MR signal changes in a turtle brain *in vitro* with GE-EPI at 9.4 T. The results of *in vivo* studies were also inconclusive. Xiong et al. succeeded in detecting magnetic fields *in vivo* and suggested the magnitude images are better than the phase images to evaluate neuronal currents [15], but Chu et al failed to reproduce the results [17]. Frequency spectral transform was performed to detect the signal related to *in vivo* alpha wave using GE-EPI, but the neuronal oscillations were not observed [19, 25]. MR techniques using the spin-lock is recently introduced to measure the oscillating magnetic field in phantom[23, 24], but Chai et al. failed to detect neuronal currents in *in vivo* imaging.

Compared with the conventional resting-state fMRI and spectral analysis, our approaches have several differences. First, the spectrum of interest was limited by TR, i.e., [$-\frac{1}{2TR},\frac{1}{2TR}$], in the previous fMRI studies [13, 16, 19, 25]. Since spontaneous neuronal oscillations have a wide frequency range (~200 Hz) [41], they may induce aliased signals in the frequency spectrum. To our knowledge, it has not been tried to analyze resting-state fMRI data in the viewpoint of the aliased neuronal signals. In the proposed method, the frequency above the Nyquist frequency could be analyzed by setting the target frequency as described above and the two-TR approach allowed us to evaluate a wide frequency range above Nyquist frequency with no *a priori* information. Second, most resting-state fMRI studies are based on magnitude images, but our phantom and simulation studies showed that complex and phase images provided much better sensitivity than magnitude images in the spectrum. Previous simulation and *in vitro* studies also showed that the phase signals are more significant than the magnitude signals in detecting the oscillating magnetic fields [44, 47]. A simulation study considering the realistic geometry and physiologic of human neuron also demonstrated that the phase signals are higher than the magnitude signals [48], although neuronal signals could be affected by many factors including spatial distribution of neurons within pixels. Third, we proved that TE should be carefully determined according to the target frequency of interest. When TE was the same as the period of the target frequency, the target signal could not be detected due to the cancellation effect (Fig 5C). TE should be chosen as half the period of the target frequency to minimize the cancellation effect or shorter to minimize the T_2_* signal decay. Therefore, our study additionally provides a new insight into evaluation of conventional (resting-state) fMRI data.

Although only two-TR experiments were demonstrated in this study, data acquisitions with more than two TRs are possible and may help improving the specificity and reliability of the absolute spectrum. Also, the two-TR acquisitions were alternated during 200 TRs in this study. The reliability in *in vivo* may be improved by changing TRs for consecutive excitations, where variations in T_1_ relaxation should be considered together.

The proposed method was validated on the phantom made of the copper wire. Although the copper wire might cause susceptibility artifacts, the static artifacts would not affect the detectability of the proposed method significantly, because it detects the dynamic change from the oscillating magnetic field.

Several confounding factors should be considered for *in vivo* imaging. First, non-neuronal noises such as systematic vibrations and physiological noises can still contaminate the peaks produced by neuronal oscillations. Second, neuronal oscillations may have signals within a wide frequency band rather than at a specific frequency, which may spread the detected signals and thus decrease SNR and the detectability. The sensitivity is also a major problem for *in vivo* imaging. Although the experiments were performed on the phantom of a long T2 value, the proposed method provided a potential solution to improve the sensitivity. We demonstrated that temporal averaging increased the sensitivity but it requires a long scan time that may induce motion and/or B_0_ drifting effects. Nonetheless, the proposed method can detect signals from weak oscillating magnetic fields with frequency above Nyquist sampling frequency in the absolute frequency spectrum with no *a priori* target frequency information. Therefore, it may help us to detect weak oscillating magnetic fields *in vivo*, which warrants further investigation.

## Conclusion

We introduced the multiple-TR approach and Fourier analysis to detect weak oscillating magnetic fields above Nyquist frequency. The approach could precisely evaluate aliased signals from oscillating magnetic fields with random frequencies and on/off intervals, which was demonstrated in both simulations and phantom studies. The detected signals showed higher SNR with increasing number of dynamic scans, demonstrating that temporal averaging is possible with the proposed approach. The two-TR approach provided the absolute frequency spectrum above the Nyquist frequency pixel by pixel with no *a priori* target frequency information. The proposed method has many advantages for detecting high frequency neuronal oscillations, which warrants further investigation.
